# Comparison of ISG15, IL28B and USP18 mRNA levels in peripheral blood mononuclear cells of chronic hepatitis B virus infected patients and healthy individuals 

**Published:** 2019

**Authors:** Seyed Mohammad Ali Hashemi, Jamal Sarvari, Mohammad Reza Fattahi, Razieh Dowran, Amin Ramezani, Seyed Younes Hosseini

**Affiliations:** 1 *Department of Bacteriology and Virology, Shiraz University of Medical Sciences, Shiraz, Iran*; 2 *Gastroenterohepatology Research Center, Shiraz University of Medical Sciences, Shiraz, Iran*; 3 *Institute for Cancer Research, Shiraz University of Medical Sciences, Shiraz, Iran *

**Keywords:** Hepatitis B viruses, Immunity, ISG15 protein, IFN-lambda-3 protein, USP18

## Abstract

**Aim:**

The purpose of this study was to evaluate the expression level of Interferon-stimulated Gene 15 (ISG15), Interleukin28B (IL28B) or IFN-lambda-3 and Ubiquitin specific peptidase 18 (USP18) genes in Peripheral Blood Mononuclear Cells (PBMCs) of patients with chronic active and inactive hepatitis B in comparison with healthy individuals.

**Background:**

Despite the presence of the vaccine for hepatitis B virus (HBV), it remains a public health challenge. The effort to uncover the immune genes attributed to infection outcome is going through.

**Methods:**

This Cross-sectional study was conducted on hepatitis B infected patients that were admitted to the Clinic of Liver diseases, Shiraz, January-November 2016. Patients were divided into two groups including active and inactive chronic regarding relevant World Gastroenterology Organization Global Guideline. They were mono-infected with HBV, and HCV or HIV co-infection was excluded from the study. Gene expression analysis was performed on fresh PBMCs samples with the help of Real-time PCR method.

**Results:**

Interleukin 28B gene expression showed no statistically significant difference between the three studied groups (P>0.05). The expression level of ISG15 was significantly higher in the healthy control group compared to active (P= 0.0068) and inactive chronic subjects (P<0.0001). Similarly, USP18 expression level in the control group was also significantly higher compared to the active (P= 0.0228) and inactive chronic patients (P=0. 0226).

**Conclusion:**

The results of this study showed that the expression level of ISG15 and USP18 but not IL28B were higher in healthy individuals than in those infected with HBV. This difference expression may highlight the role of ISG15 and USP18 in the immune-related mechanism of HBV infection.

## Introduction

 Despite the availability of an effective vaccine and advanced treatment options for hepatitis B virus (HBV), elimination is yet to be achieved in the world. Recent findings indicated that the rate of inactive carrier has significantly reduced among young and children by vaccination (1,2). However, about 250 million people around the world are chronically infected with the virus (3). The prevalence of HBV infection in Iranian people is estimated at 2-7% (4). It is estimated that up to 30% of Liver Cirrhosis and 50% of Hepatocellular Carcinoma are attributed to HBV infection (5). 

Acute hepatitis B infection usually clears spontaneously by the strong complex response of the immune system, including neutralizing antibody secretion and Kupffer, NK, CD4^+^ and CD8^+^cells activities concomitant with IL-2, Interferon gamma/alpha and tumor necrosis factor (TNF) production (6). Some people are unable to resolve the acute infection, so progress towards chronicity status (7). The patients with persistent infection are amenable for subsequent progression toward cirrhosis and even liver cancer. 

As HBV was not determined to be cytolytic, host immune reaction to infected hepatocyte is accepted as the major causes of chronic damage of the affected liver (8). Apparently, it could be extrapolated that immune reaction ascertains the fate of HBV chronic infection, the extent of pathogenicity and disease outcome (9). In the recent studies, various genes that involved in antiviral defense and immune-pathogenesis of hepatitis has been considered. Among these, Interferon-stimulated Gene 15(ISG15), Interleukin 28B (IL28B) and Ubiquitin specific peptidase 18 (USP18) remarkably were attained special attention (10-13).

Interferon-stimulated gene 15 which is triggered by type I interferon, exhibits an innate antiviral effect. Although a number of studies have shown that ISG15 perform an antiviral activity to hepatitis C virus (12, 14), others reported its enhancing role for virus replication (15). Kim *et al*. demonstrated that HBV replication, is not affected by the ISGylation activity of ISG15 (16) and another study by Qiu *et al*. indicated that the expression level of ISG15 in HBV liver tumor is higher than normal liver cells (17).

From recent findings, polymorphism of IL28B gene at rs12979860 has shown to correlate with IFN-treatment response as well as spontaneous clearance of HCV (18-20). Similarly, Sonneveld *et al*. reported that in HBeAg^+^ chronic patients receiving IFN therapy, a significant correlation between IL28B polymorphism and HBe seroconversion or HBs clearance has been determined (21). In the case of IL28B expression, Li *et al. *suggested a possible role for IL28B as its level was decreased among chronic hepatitis B patients compared to resolved individuals (11).

Ubiquitin specific peptidase 18 which is also known as UBP43 is an enzyme belonging to the specific ubiquitin proteases (22). The protein specifically cleaves ISG15 protein thus would destroy its functions. Therefore, USP18 plays its own roles in the innate immune system through pre-defined activities of ISG15(13). The defined interaction of ISG15 and USP18 has an important impact in the innate immune response against viral infections such as hepatitis viruses (13).

The role of immune genes in the chronic hepatitis B disease process is under investigation, due to the controversial issue. Regarding studies, on the other viruses, probably a different immune gene expression such as (ISG15) gene, (IL28B) and (USP18) be impressive in the HBV infection progress and outcome (10, 11, 23). The expression analysis of these genes (ISG15, IL28B, and USP18) may be beneficial in the patient management as introduce new predictive markers of disease progression. Therefore, the purpose of this study was to evaluate the expression level of these genes in Peripheral Blood Mononuclear Cells (PBMCs) of HBV infected patients with chronic active and the inactive carrier in comparison to healthy individuals. 

## Methods

**Study population**

This Cross-sectional study was conducted on hepatitis B infected patients that referred to Motahhari Clinic of Gastrohepatointestinal, Shiraz, Iran between January 2016 and November 2016 (11 months). Patients were divided into two groups including active and inactive chronic regarding relevant World Gastroenterology Organization Global Guideline (24). In this regard, the inactive carrier group consisted of chronic patients, which had less than 2000 IU / ml viral load and normal liver enzyme level. The active chronic group consisted of chronic patients who had a viral load of over 2000 IU/ml up to 10^9^ and elevated liver enzymes more than 2-3 times of normal level. 

Groups of active chronic and inactive carrier patient who had medical records at Liver Clinic (Motahhari Clinic of Gastrohepatointestinal Diseases affiliated to Shiraz University of medical sciences) were enrolled in the study based on clinical and Laboratory records. They were mono-infected with HBV, and HCV and HIV co-infection excluded from the study. Finally, 27 inactive carrier patients, and 18 active chronic patients selected from data included in the study. A healthy control group, including 10 age and gender matched individuals were also enrolled.

All sampling steps performed on participants with written consent form patients and respect to the ethical standards of the Shiraz University of Medical Sciences as well as with the 1964 Helsinki Declaration. Ethics Committee of Shiraz University of Medical Sciences approved the study and informed consent was obtained from all participants (Ethic code: IR.SUMS.REC.1394.S1137).

**PBMC isolation/RNA extraction and Real-time PCR**

Fresh PBMCs of all patients and healthy subjects were isolated from total blood using ficoll gradient (Lymphodex, Norway, Oslo) regarding recommended instruction. Then, approximately 10^6 ^cells introduced into total RNA extraction using Trizol^TM^ (RNx plus^TM^, Cinnagen Inc., Iran) and quality/quantity of RNA was assessed through Nanodrop^TM^ spectrophotometry as well as gel electrophoresis analysis. The cDNA was then synthesized with the help of an RT-PCR kit (Thermo Fisher Scientific Inc. USA) considering protocol by using one microgram RNA. In this project, the gene expression of ISG15, IL28 and USP18 was carried by SYBR green based Real-time PCR and a 7500 Real-Time PCR System (Applied Biosystems, Grand Island, NY, USA). The volume of prepared reaction was 25 ml and each reaction contained 12.5 μL 2x master mix green (Ampliqon Inc., Denmark) with high Rox passive reference dye, 0.5 μL each primer (TAG Copenhagen A/S, Denmark ), 2 μL of cDNA and 9.5 μL water. The PCR program was started by a denaturation step at 95° C for 15 min followed by 40 cycles of 95° C for 15 s and annealing / extension at 6° C for 1 min. β-actin gene was also employed as a the reference gene to normalize the results. At the end, to calculate the relative expression of each gene, the 2^-ΔCt^ method was applied for each sample cycle threshold. Primer design carried out with AlleleID version7 software and with the help of NCBI database. Primer sequences are shown in Table 1.

**Statistical analyses**

The data normality was carried out by using the Shapiro-Wilk test. According to the fact that the data were not normal, the Kruskal-Wallis test used to compare the three groups. In other hand Mann-Whitney U used for pairwise comparisons. P <0.05 was considered as meaningful. In this study, for statistical analysis GraphPad Prism^TM^ version 5 was employed. 

## Results

**Patients**

In this study 27 inactive carrier subjects, 18 active chronic patients and 10 healthy control individuals were enrolled. Demographic and clinical data for the patient groups are shown in Table 2.

**Table 1:** The sequences of primer sequences which were used in this study

**Gene **		**Sequence (5'->3')**	**Product length**
β-Actin(44)	Forward Primer Reverse Primer	5'-GCCTTTGCCGATCCGC-3' 5'-GCCGTAGCCGTTGTCG-3'	90bp
USP18	Forward Primer Reverse Primer	5'-GACTCCTTGATTTGCGTTG-3' 5'-TTGCTTGATAACTCCCTGG-3'	155bp
IL28	Forward Primer Reverse Primer	5'-CCACATAGCCCAGTTCAA-3' 5'-GAAGCGACTCTTCTAAGG-3'	83bp
ISG15(44)	Forward Primer Reverse Primer	5'-TCATCTTTGCCAGTACAGGAGC-3' 5'-TTCTGGGTGATCTGCGCCTT-3'	160bp

**Table 2:** Demographic and clinical data for the Patient groups

**Variables **	**chronic active**	**inactive chronic**
Gender		
Male	10	21
Female	8	6
Age (Mean± SD)	35.4±11.6	48.7±12.2
Laboratory test		
ALT*(Mean± SD)	43.1±36.9	25.7±9.2
AST*(Mean± SD)	41.1±35.6	23.2±4.4
ALP*(Mean± SD)	187±81.7	208.7±77
Viral load	>2000IU/ML	<2000IU/ML

* ALT: Alanine Aminotransferase; AST: Aspartate Aminotransferase; ALP: Alkaline phosphatase

**Analysis of IL28B expression among three studied groups**

**Figure 1:**
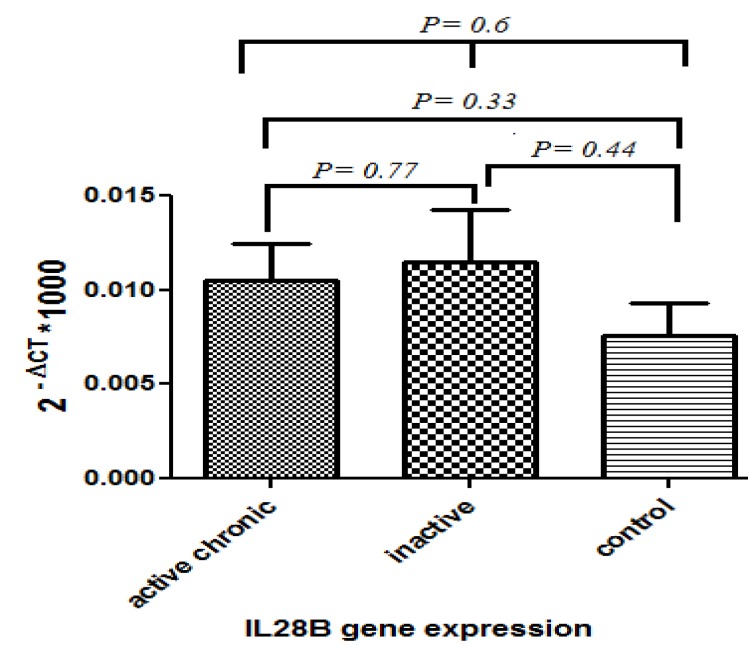
IL-28B Gene expression in PBMC of chronic active and inactive carrier hepatitis B infected patients as well as healthy individuals control group. NO significant differences were detected among groups

The real-time PCR analysis of gene expression revealed that our selected genes did not show resembling results in three study groups. Interleukin 28B gene expression analysis showed a complete different pattern from other two genes (Figure 1). In this case, in spite of our expectation, significant differences were not detected among groups. Since there are 96% similarities between IL28A and IL28B at the DNA level, there is a potential for cross-reactivity in laboratory tests.

**Figure 2:**
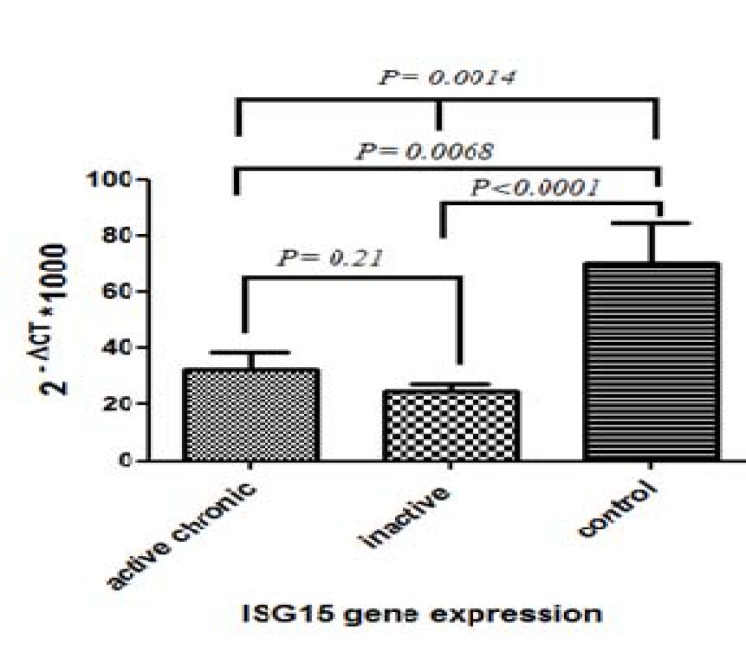
ISG15 Gene expression in PBMC of chronic active and inactive carrier hepatitis B infected patients as well as healthy individuals control group. A statistically significant decrement of gene expression was detected in patient groups compared to healthy individual

**Figure 3:**
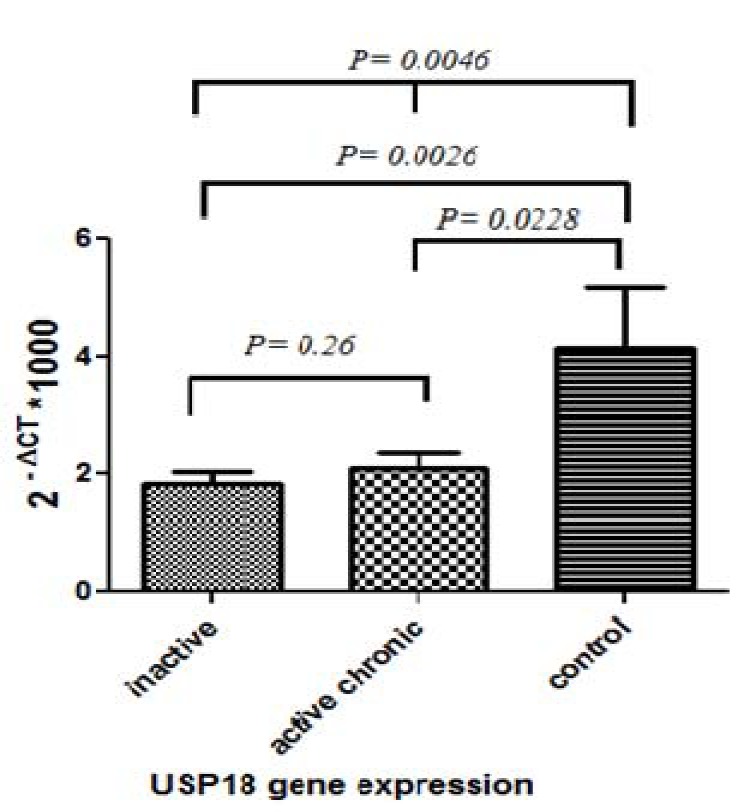
USP18 Gene expression in PBMC of chronic active and inactive carrier hepatitis B infected patients as well as healthy individuals control group. A statistically significant decrement of gene expression was detected in patient groups when compared to healthy individual

**Analysis of ISG15 and USP18 expressions among groups**

 In contrast to IL28B, ISG15 expression level was significantly higher in the healthy group in comparison with both active and inactive chronic infected patients. Moreover, when active and inactive chronic groups were compared together, no significant difference in their expression levels was detected (Figure 2).

Similarly, USP18 expression mimics ISG15 expression pattern in different groups. Its expression showed a significant higher level for healthy group when compared to HBV infected patients. Also, in a same trend active and inactive chronic groups exhibited resemble expression with no significant difference as shown in figure 3.

Figure 3 **.** USP18 Gene expression in PBMC of chronic active and inactive carrier hepatitis B infected patients as well as healthy individuals control group. A statistically significant decrement of gene expression was detected in patient groups when compared to healthy individual.

## Discussion

The Persistent infection of HBV can progress to cirrhosis and eventually to liver cancer (25). Efforts to uncover immune genes attributed to chronicity, infection outcome and disease progression of HBV are going through. Uncovering Immune genes responsible for disease progression and disease outcome could be beneficial for their well-defined management. In this study, the possible role of three innate immune genes in the disease process investigated among active chronic, inactive chronic (immune control phase) and healthy control groups. 

Although the prognostic role of IL28B variants and expression has been determined in the process of hepatitis C virus treatment as well as clearance, similar studies regarding its importance in HBV are limited and ambiguous (19, 26). Interestingly, in this study, no significant difference in IL28B expression was detected in PBMCs among three different study groups. As the result showed a similar pattern even in healthy and infected groups, it may highlight no important role of this gene at least in circulating PBMCs for disease progression. In a similar study, Peng *et al.*, have shown that IL28B polymorphism has no significant effects on the outcome of hepatitis B infection (27). Hence, another study has acclaimed that IL28B variation just affects the immune response to HCV but not HBV when investigated in 226 chronic HBV patients (27, 28). However, another study demonstrated a possible role of this gene in disease progression as a vague idea that depends on HBeAg status (29). Rangnekar *et al.*, in a meta-analysis study, showed IL-28B genotype is significantly related with SVR in HCV genotype 1 patients of varying race, receiving pegIFN and ribavirin (26).

In contrast, reports regarding a tight relation between IL28B genotypes and disease progression, including viral load, liver inflammation, and cancer risk is growing recently (11, 30). It has been shown that rs10853728 CC genotype is related to the higher liver inflammatory activity. From this, it was suggested that in HBeAg-negative patients, IL28B can play a role through stimulation of innate immune response (29). Li *et al.* demonstrated that serum levels of IL28B are lower in patients with chronic hepatitis B than resolved individuals, emphasizing the role of this cytokine in virus clearance (11). 

The role of ISG15 and its enzymatic properties in different viral defense have recently been studied (31, 32). In our study, ISG15 gene expression in healthy group was significantly higher than in chronically infected patients. Albeit, there was no significant difference between active and inactive infected individual groups for ISG15 expression. The report considers that the anti-viral role of ISG15 has not met a consensus idea yet and the gap needs more investigation to be filled. For some viruses like VSV and LCMV, it demonstrated that ISG15 action is redundant as an antiviral defense for mouse model (33). Even more, in an HCV-related experiment, ISG15 determined to act as an enhancer molecule for further virus spread (34). In contrast, ISG15 deficient mice demonstrated to be more sensitive to influenza and herpes simplex virus infections (31). A series of studies have shown that ISG15 has an impressive antiviral activity to hepatitis C virus (15). In the case of HBV infection, results are also controversial. While Kim *et al.* demonstrated that HBV replication did not affect the ISGylation activity of ISG15 (16), another study by Qiu *et al.* demonstrated that the expression level of ISG15 in HBV liver tumor is higher than normal liver cells (17). Just in spite of this, the recent effort was demonstrated that chronic HBV infection leaves a profound suppressive impact on liver innate immune responses by decreasing ISG15 (35). Furthermore, more recently Hoan *et al*. reported that ISG15 level in serum was higher in HBV infected patients when compared to control and they also showed an expression correlation of ISG15 with HBV-related liver diseases (36). However, Speer *et al.* showed that interestingly ISG15 exhibits critical immune functions, but not in antiviral defense of human (37). In sum, our data emphasized the poor discriminative value of ISG15 among inactive and active infected patients with HBV. 

Ubiquitin specific peptidase 18 gene which encodes a specific protease acting against ISG15 function is assigned among important modulator of antiviral defense (22, 38). It suppresses the JAK/STAT signaling pathway so ameliorate innate immune induction (39). Our results showed that its expression level in the healthy subjects was significantly higher than the inactive chronic group that highlighted the role of the virus in increasing the USP18 gene expression. Similar results were also reported in the modulatory role of USP18 in HBV induced immune response. Li *et al*. demonstrated that USP18 deficient Hep-G2 cell significantly overwhelm HBV replication through increased IFN stimulated genes (39). In the other word, the absence of USP18 suppressive action resumes the JAK/STAT signaling pathway and consequent immune induction (39). The study regarding enhancing the role of USP18 expression in HBV replication also presented in a mouse model of HBV infection (40). However, to the best of our knowledge, no report considering the relation of USP18 gene expression and disease outcome has published yet. 

In our study, the USP18 and ISG15 gene expressions are decreased in the HBV-affected patient group. The proposed reason behind this finding is the exhausted immune response during persistence infection. This phenomenon has been observed in other studies related to chronic hepatitis C (41). Inconsistent with this result, different studies demonstrated that IFN related genes, including TRIF, RIG-1, MDA5, STING decreased significantly in HBV patients while some genes like IRF-3 did not exhibit this pattern (42, 43). In overall, it seems that the expression of IFN-inducible genes was impaired in the PBMCs from HBV infected patients during persistent infection. According to our results, reduction of ISG15 and USP 18 gene expression in the chronic phase of infection suggested their direct role in virus immunity and importance. On the other side, our study showed that ISG15 and USP18 gene expression in chronic active and inactive carrier was similar that is indicative of their poor discriminative value among infected groups as well as disease outcome prediction. Our sample size completely included with the pure genotype D of the virus, regarding medical records, but the small size of the population may limit more conclusion and an investigation with bigger sample size either on more evaluated genes is demanding. Also, the genotype of IL-28B was not considered in this study. It was so valuable if we have this data in the manuscript, but due to some technical limits, it was not possible at the moment. 

The results of our survey indicated that the expression level of ISG15 and USP18 but not IL28B were higher in healthy individuals than in those infected with HBV. This difference in expression may highlight the role of ISG15 and USP18 in the immune- related mechanism of HBV infection. Moreover, we did not find significant differences between expression levels of ISG15, IL-28B and USP18 among inactive and active groups. It seems that the evaluation of gene expression in the PBMCs sample is not enough to make a reasonable relationship between immune genes and the disease outcome. More investigation of other immune system genes is suggested.
